# An Examination of Brain Abnormalities and Mobility in Individuals with Mild Cognitive Impairment and Alzheimer's Disease

**DOI:** 10.3389/fnagi.2017.00086

**Published:** 2017-04-05

**Authors:** Barbara L. Fischer, Rhonda Bacher, Barbara B. Bendlin, Alex C. Birdsill, Martina Ly, Siobhan M. Hoscheidt, Richard J. Chappell, Jane E. Mahoney, Carey E. Gleason

**Affiliations:** ^1^Geriatric Research, Education, and Clinical Center, William S. Middleton Memorial Veterans HospitalMadison, WI, USA; ^2^Department of Statistics, University of Wisconsin-MadisonMadison, WI, USA; ^3^School of Medicine and Public Health, University of Wisconsin-MadisonMadison, WI, USA; ^4^Wisconsin Alzheimer's Disease Research CenterMadison, WI, USA; ^5^Departments of Biostatistics and Medical Informatics, School of Medicine and Public Health, University of Wisconsin-MadisonMadison, WI, USA; ^6^Division of Geriatrics, Department of Medicine, School of Medicine and Public Health, University of Wisconsin-MadisonMadison, WI, USA

**Keywords:** Alzheimer's disease, mild cognitive impairment, mobility, neuroimaging, Timed Up and Go test

## Abstract

**Background:** Mobility changes are concerning for elderly patients with cognitive decline. Given frail older individuals' vulnerability to injury, it is critical to identify contributors to limited mobility.

**Objective:** To examine whether structural brain abnormalities, including reduced gray matter volume and white matter hyperintensities, would be associated with limited mobility among individuals with cognitive impairment, and to determine whether cognitive impairment would mediate this relationship.

**Methods:** Thirty-four elderly individuals with mild cognitive impairment (MCI) and Alzheimer's disease underwent neuropsychological evaluation, mobility assessment, and structural brain neuroimaging. Linear regression was conducted with predictors including gray matter volume in six regions of interest (ROI) and white matter hyperintensity (WMH) burden, with mobility measures as outcomes.

**Results:** Lower gray matter volume in caudate nucleus was associated with slower speed on a functional mobility task. Higher cerebellar volume was also associated with slower functional mobility. White matter hyperintensity burden was not significantly associated with mobility.

**Conclusion:** Our findings provide evidence for associations between subcortical gray matter volume and speed on a functional mobility task among cognitively impaired individuals.

## Introduction

Mobility limitations are concerning among geriatric patients, and may herald significant declines in health and quality of life. Associated with increased risk of falls, mobility limitations may represent the first warning sign of future loss of independence. Cognitive impairment confers additional risk for mobility limitations, and is correlated with increased levels of morbidity and mortality (Buchner and Larson, 1988). Thus, identifying and addressing individual contributors to mobility limitations is critical in older adults with cognitive decline.

Examination of contributing factors has recently focused on abnormalities in brain structure. Subclinical brain abnormalities have been documented in association with mobility limitations and falls in cognitively intact older individuals (Rosano et al., 2007, 2008; Callisaya et al., 2009; Soumaré et al., 2009; Srikanth et al., 2009), and associations have been observed between white matter changes and very mild cognitive decline (Smith et al., 2015). Associations have also found between white matter integrity and gait parameters among cognitive intact elders (Baezner et al., 2008; de Laat et al., 2011). However, few studies have investigated such abnormalities in patients with cognitive impairments. Among these, preliminary research indicates subtle differences in both white and gray matter structure. For example, Onen et al. (2008) found an inverse relationship between white matter abnormalities and mobility performance in older adults with mild cognitive impairment (MCI). Evidence of cortical abnormalities has also been observed. Specifically, among individuals with MCI, smaller volume in the primary motor cortex was associated with slower single and dual task gait and greater gait variability (Annweiler et al., 2013), reduced superior and middle frontal lobe gray matter volume was associated with falls (Makizako et al., 2013), and temporal lobe atrophy was associated with slower gait speed (Makizako et al., 2011).

While these preliminary studies suggest that a variety of brain abnormalities can contribute to mobility limitations and falls, the evidence to date has been sparse, and it is unclear to what extent severity of illness influences the presentation of mobility limitations. For example, few studies have examined individuals across a range of cognitive impairment, including MCI and dementia. Moreover, within this population, even fewer studies have investigated both white and gray matter abnormalities together to examine the relative contributions of each. We aimed to fill this gap in the literature by examining the relationship between neuroanatomical variables (e.g., white matter lesion burden and regional gray matter volume) and mobility and falls in cognitively impaired older adults. Hypothesizing that structural abnormalities would be associated with mobility limitations across a spectrum of cognitive decline, we assessed individuals with a range of cognitive impairment. Additionally, since cognition has been shown to correlate with mobility (e.g., Gleason et al., 2009), we further hypothesized that cognition, specifically executive function, would mediate the relationship between structural brain abnormalities and mobility limitations.

## Materials and methods

### Participants

Thirty-four individuals who had been previously diagnosed with either MCI due to presumed Alzheimer's Disease (AD) pathology (according to Petersen's (2004) criteria) or AD were recruited for participation in the study from the William S. Middleton Memorial Veterans Hospital and the University of Wisconsin-Madison Alzheimer's Disease Research Center (Wisconsin ADRC). Patients underwent neuroimaging prior to study entry, and all participants were reviewed at a multidisciplinary diagnostic consensus panel. This study was carried out in accordance with the recommendations of guidelines on human experimentation from the Human Participants Research Committee, with written informed consent from all subjects. All subjects gave written informed consent in accordance with the Declaration of Helsinki. The protocol was approved by the University of Wisconsin-Madison IRB.

We enrolled patients with a range of cognitive impairment to investigate how brain abnormalities across a spectrum of cognitive impairment would affect mobility. We calculated that sample sizes of 25 participants per group would be sufficient to detect significant differences in mobility parameters and cognitive performance between individuals with MCI and AD. Given that all participants were thought to share a common underlying AD pathology, when we were unable to recruit 50 participants, we combined individuals into one group of 34.

Individuals were eligible to participate if they were 65 years of age or older, had a study partner accompanying them on a study visit, were English speaking, and were able to provide informed consent. Exclusion criteria included: (a) presence of a central neurological disorder other than AD or MCI, identified on clinical examination prior to study entry, that could account for limited mobility or cognitive deficits; (b) abnormal findings on any previous MRI other than global atrophy and incident white matter changes; (c) use of medications affecting cognition and mobility within 3 days of evaluation (anti-psychotics tricyclic anti-depressants, benzodiazepines, and other sedatives); (d) use of any high dose anti-depressants (dose at or above highest level for geriatric dosing guidelines); (e) contraindications for MRI scanning (claustrophobia, metal implants); (f) ongoing significant substance abuse (American Psychiatric Association, 2000); (g) inability to safely walk a short distance (assistive devices allowed); (h) inability to understand study instructions; and (i) severe visual impairment (inability to read a newspaper with corrective lenses).

### Cognitive and mood measures

All participants underwent a comprehensive battery of cognitive and tests. These included the Mini-Mental State Examination (Folstein et al., 1975) the Stroop Color-Word Test (Golden, 1978) Trail Making Tests A and B, (Partington and Leiter, 1949) Wechsler Adult Intelligence Scale, Fourth Edition (WAIS-IV) Digit Span and Digit Symbol subtests (Wechsler, 2008) Wechsler Memory Scale, Third Edition (WMS-III) Letter Number Sequencing subtest (Wechsler, 1997) and semantic (Animals, Fruits, Vegetables; Goodglass and Kaplan, 1972) and phonemic fluency (FAS), (Strauss et al., 2006) as well as the Geriatric Depression Scale-Short Form (Fountoulakis et al., 1999).

### Principal components analysis

A principal components analysis (PCA) was conducted on 16 correlated cognitive variables: time to complete TMT A, time to complete TMT B, number of words read in 45 s for each of the Stroop Test conditions (i.e., Word, Color, Color-Word), LNS total score, Digit Symbol (total number of correctly trascribed numbers), Digit Span (length Forward and Backward), Digit Span Total points, and phonemic (F, A, S) and semantic (animal, fruit, and vegetable) fluency (total number of words generated). Because higher scores for TMT A and B represented poorer performance, their values were reversed. The first component explained 43.8% of the variability, while all other components accounted for far less variance. A linear combination of all 16 variables (centered and scaled) created one composite executive function score in which larger values represented poorer executive function. Five variables each contained one missing value; these were imputed using regression imputation against the remaining complete variables (Little and Rubin, 2002). Results were not qualitatively different when missing values were imputed with mean values.

### Mobility measures

Participants were administered the Timed Up and Go test (TUG) in single and dual task format (TUG-alone and TUG-cog; Podsiadlo and Richardson, 1991; Shumway-Cook, 1997), as follows. The examiner demonstrated the TUG-alone. Participants were then asked to perform the TUG task independently: first alone, and second with a cognitive task (e.g., reciting the days of the week in reverse order). This task has been utilized previously to increase cognitive load on the TUG (Kelly et al., 2008). Participants were also administered the Dynamic Gait Index (DGI) (Guralnik et al., 1995). Total score on this test has been shown to demonstrate acceptable concurrent validity with other similar measures, as well as adequate test–retest and inter-rater reliability (Jonsdottir and Cattaneo, 2007). Further details on TUG and DGI administration are provided in the Supplemental Material.

### Brain imaging

MR scanning was performed on a General Electric 3.0 Signa HDxt MRI scanner (General Electric, Milwaukee, WI) with an 8-channel head coil. All participants underwent a similar 3D T1-weighted scan with acquisition parameters: TI = 450 (or 900) ms; TR = 7.79 (or 6.62) ms; TE = 2.98 (or 2.84) ms; flip angle = 12° (or 8°); acquisition matrix = 256 × 256 mm, FOV = 256 mm; slice thickness = 1.0 (or 1.2) mm, no gap. A high resolution 3D T2-weighted fluid attenuated inversion recovery (FLAIR) scan was collected with acquisition parameters: TI = 1,907 (or 1,905, or 1,874) ms; TR = 6,200 ms; TE = 125.37 (or 126.06, or 140.98) ms; flip angle = 90°; acquisition matrix = 256 × 256, FOV = 256 mm; slice thickness = 1.2 mm, no gap. All images were reviewed by a neuroradiologist.

#### White matter hyperintensities

In order to determine white matter hyperintensity (WMH) volume, we used the Lesion Segmentation Tool (LST) implemented in SPM8 (Wellcome Trust Centre for Neuroimaging, Institute of Neurology, UCL, London UK, http://www.fil.ion.ucl.ac.uk/spm). Lesions are seeded based on spatial probability from T1 images and hyperintense outliers on T2FLAIR images. The initial threshold was set at 0.30 and is used to create the binary conservative lesion belief map from the gray matter lesion belief map. Next, a growth algorithm grew seeds from the conservative lesion belief map toward a probabilistic liberal lesion belief map from gray matter, white matter, and cerebrospinal fluid. Lastly, we used a threshold of 1.00 on the resulting lesion belief map. Intracranial volume (ICV) was calculated to scale for differences in head size in the WMH analyses using a reverse brain masking method implemented in SPM8. The resulting WMH volume was divided by ICV to give a ratio (WMHr). WMHr-values were further analyzed using statistical modules implemented in R (R Development Core Team, 2008). Please see Ithapu et al. (2014) for a description and statistical analysis of the lesion segmentation tool.

#### Gray matter volume

Processing of the T1-weighted images was performed using a six-class segmentation processing stream in SPM8 (Wellcome Trust Centre for Neuroimaging, Institute of Neurology, UCL, London UK, www.fil.ion.ucl.ac.uk/spm). Processing involved bias correction and iterative normalization and segmentation of the original anatomic images into distinct tissue classes (gray matter, white matter, cerebrospinal fluid, skull, fat tissue, and image background) using spatial prior information. Gray matter tissue segments were normalized to Montreal Neurological Institute (MNI) template space via a 12-parameter affine transformation and non-linear deformation (with a warp frequency cutoff of 25). The segmented and normalized gray matter maps were “modulated,” which involves scaling the final gray matter maps by the amount of contraction or expansion required to warp the images to the template. The normalized, segmented, and modulated gray matter images were smoothed using an 8 mm full width at half maximum (FWHM) Isotropic Gaussian Kernel. The final result was a gray matter probability map for each participant in which the total amount of gray matter remained the same as in the original images.

**Figure 1:**
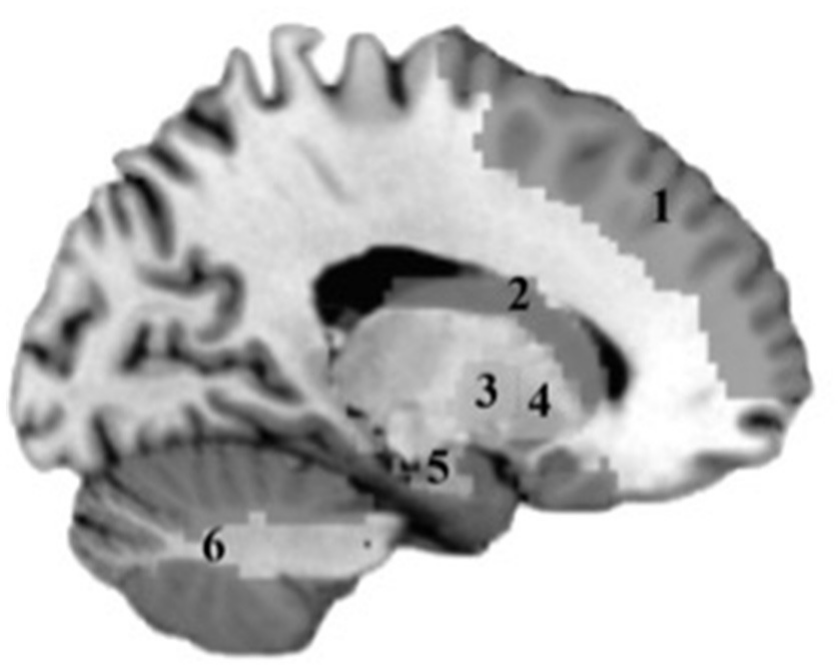
**Gray matter regions tested in analyses**. 1, Frontal cortex; 2, Caudate; 3, Putamen; 4, Globus Pallidus; 5, Hippocampus; 6, Cerebellum.

Mean bilateral gray matter regions of interest (ROIs) were calculated for the following regions: caudate, cerebellum, frontal (encompassing inferior, middle, and superior gyri), globus pallidus, hippocampus, and putamen. These regions were selected based on their known contributions to motor function. Standardized anatomically based ROIs were generated using the labels of the Anatomic Automatic Labeling (AAL) system implemented in the Wake Forest PickAtlas (WFU) toolbox (Lancaster et al., 2000; Tzourio-Mazoyer et al., 2002; Maldjian et al., 2003) in SPM8 (Wellcome Trust Centre for Neuroimaging, University College, London). The following AAL parcellations were used: caudate [Caudate_L, Caudate_R], cerebellum [Cerebellum_3_L, Cerebellum_3_R, Cerebellum_4_5_L, Cerebellum_4_5_R, Cerebellum_6_L, Cerebellum_6_R, Cerebellum_7b_L, Cerebellum_7b_R, Cerebellum_8_L, Cerebellum_8_R, Cerebellum_9_L, Cerebellum_9_R, Cerebellum_10_L, Cerebellum_10_R], frontal [Frontal_Sup_L, Frontal_Sup_R, Frontal_Mid_L, Frontal_Mid_R, Frontal_Inf_Oper_L, Frontal_Inf_Oper_R, Frontal_Inf_Orb_L, Frontal_Inf_Orb_R, Frontal_Inf_Tri_L, Frontal_Inf_Tri_R], globus pallidus [Pallidum_L, Pallidum_R], hippocampus [Hippocampus_L, Hippocampus_R], putamen [Putamen_L, Putamen_R]. The volumetric mean (mm^3^) of each gray matter ROI was extracted for each subject. Gray matter ROI are shown in Figure 1.

### Statistical analysis

Linear regression analyses were implemented for outcomes: DGI total, TUG alone, TUG cognition, and executive function composite score. The predictors of interest were white matter lesion burden and the six gray matter volume ROIs: frontal cortex, hippocampus, putamen, caudate, globus pallidus, and cerebellum. White matter hyperintensities were log transformed to adjust for an extreme value in one patient's total white matter lesion burden. The six gray matter ROIs were also log transformed. All predictors were divided by ICV giving a ratio. All tests included age and were adjusted for multiple testing separately within each of the four outcomes (DGI total, TUG alone, TUG cognition, and executive function composite score) using the Bonferroni correction. Analyses were performed using the R programming language, version 3.0.1.

## Results

### Demographics and baseline cognition

Baseline characteristics of the sample are presented in Table 1. Study participants were elderly (mean age 78.4 years old), well-educated (mean education 15.6 years), and predominantly male (68%). Global cognition for the sample was mildly to moderately impaired ($\bar{X}$ MMSE = 24.6). Seventeen participants were diagnosed with MCI and 17 with AD. Average WMHr $\bar{X}$ was 1.1 ml/100 sd 1.3 ml/100. Due to sample size considerations, individuals with MCI and AD were combined into one group in further analyses.

**Table 1:** **Baseline characteristics and test results**.

**Subject characteristics**	**Mean (standard deviation)**
**DEMOGRAPHICS**	
Age (years)	78.4 (6.1)
Education (years)	15.6 (2.9)
Gender (N, % female)	11 (32)
Diagnosis (N, % with MCI)	17 (50)
**NEUROPSYCHOLOGICAL PARAMETERS**	
MMSE (total score out of 30 possible points)	24.6 (3.4)
WRAT-Read (total score out of 70 possible points)	32.3 (6.1)
Stroop Word (total words read in 45 s)	76.6 (18.0)
Stroop Color (total colors read in 45 s)	49.2 (13.5)
Stroop Color-Word (total words read in 45 s)	21.6 (10.6)
Trail Making A (total time to complete in s)	75.1 (111.6)
Trail Making B (total time to complete in s)	196.6 (133.0)
Digit Symbol (total digits completed in 90 s)	38.2 (13.0)
Digit Span (total score out of 48 possible points)	14.8 (4.1)
Letter Number Sequencing (total score of 30 possible points)	6.2 (2.8)
Verbal Fluency (total number of words produced in 60 s)	
Animals	12.8 (5.5)
Vegetables	6.8 (2.9)
Fruit	8.7 (4.6)
F	12.2 (4.1)
A	8.9 (4.0)
S	12.2 (4.2)
Mobility parameters	
TUG-alone (time to complete in s)	11.7 (3.9)
TUG-cog (time to complete in s)	22.9 (12.1)
Dynamic Gait Index (total score out of 24 possible points)	18.5 (3.2)
MRI parameters	
WMHr [White Matter Hyperintensity ratio (WMH/intracranial volume)]	1.1 (1.3)

*MMSE, Mini-Mental State Examination; WRAT-R Read, Wide Range Achievement Test-Revised, Reading subtest; s, second; WMHr, White Matter Hyperintensity ratio (WMH/intracranial volume).; TUG, Timed Up and Go; Instructions for administration of the TUG tasks and Dynamic Gait Index are provided in the Supplemental Material*.

### White matter hyperintensities

WMHr was not significantly associated with mobility measures or with executive function (Table 2). This precluded a mediation analysis of white matter, executive function, and gait.

**Table 2:** **Linear regression models for (A) executive function and DGI total outcomes, (B) TUG alone and TUG cog outcomes**.

**Model**	***P*-value (adjusted)**	***P*-value (unadjusted)**	**Estimate**	**95% Confidence Interval (adjusted)**	**Correlation**
**(A)**					
**EXECUTIVE FUNCTION**					
Log white matter lesions	1.00	0.23	0.53	(−0.73, 1.80)	0.28
Log putamen	1.00	0.35	−3.53	(−14.36, 7.30)	−0.15
Log hippocampus	1.00	0.52	4.44	(−15.22, 24.11)	0.21
Log globus pallidus	1.00	0.22	−3.44	(−11.46, 4.57)	−0.22
Log frontal	1.00	0.20	−12.25	(−39.30, 14.81)	−0.29
Log cerebellum	1.00	0.89	1.03	(−20.15, 22.21)	0.05
Log caudate nucleus	0.34	0.05	−10.40	(−24.96, 4.16)	−0.25
**DGI TOTAL**					
Executive function	0.61	0.08	−0.30	(−0.78, 0.18)	−0.36
Log white matter lesions	1.00	0.60	−0.22	(−1.44, 1.00)	−0.34
Log putamen	1.00	0.40	2.97	(−7.29, 13.22)	0.08
Log hippocampus	1.00	0.41	−5.30	(−23.79, 13.18)	−0.45
Log globus pallidus	1.00	0.45	2.01	(−5.67, 9.69)	0.13
Log frontal	1.00	0.64	4.21	(−21.96, 30.38)	0.29
Log cerebellum	1.00	0.50	−4.60	(−24.46, 15.25)	−0.18
Log caudate nucleus	1.00	0.27	5.46	(−8.94, 19.85)	−0.09
**(B)**					
**TUG ALONE**					
Executive function	0.86	0.11	0.38	(−0.30, 1.06)	0.33
Log white matter lesions	1.00	0.75	0.19	(−1.51, 1.89)	0.23
Log putamen	1.00	0.71	1.84	(−12.59, 16.26)	0.09
Log hippocampus	1.00	0.78	2.55	(−23.49, 28.59)	0.28
Log globus pallidus	1.00	0.39	3.18	(−7.5, 13.86)	0.12
Log frontal	0.97	0.12	−18.99	(−54.16, 16.18)	−0.39
Log cerebellum	0.02	0.00	26.45	(2.44, 50.45)	0.51
Log caudate nucleus	1.00	0.33	−6.80	(−26.95, 13.36)	0.01
**TUG COG**					
Executive function	0.02	0.00	2.13	(0.22, 4.05)	0.53
Log white matter lesions	1.00	0.40	−1.62	(−7.20, 3.96)	0.06
Log putamen	1.00	0.87	2.69	(−45.20, 50.57)	0.05
Log hippocampus	1.00	0.49	20.19	(−65.50, 105.87)	0.33
Log globus pallidus	1.00	0.26	13.77	(−21.28, 48.82)	0.17
Log frontal	1.00	0.21	−51.55	(−169.64, 66.54)	−0.35
Log cerebellum	1.00	0.18	41.84	(−47.77, 131.46)	0.27
Log caudate nucleus	0.35	0.04	−45.31	(−108.63, 18.02)	−0.14

*Each predictor lists the p-value (adjusted within model and unadjusted), the estimated effect, and the adjusted 95% confidence interval. Pearson's correlation is also calculated between the outcome and each predictor*.

### Cognition and mobility

After controlling for age, the Executive Function composite score was associated with performance on TUG cog, with stronger executive function (e.g., smaller numbers) correlated with faster TUG cog speed (Table 2; *p* < 0.01). A one unit increase in the executive function score is estimated to increase the TUG cog scores by 2.13 units and the 95% adjusted confidence interval for this change is (0.22, 4.05).

### Gray matter volume

After correcting for ICV, higher gray matter volume in the caudate nucleus was associated with faster performance on the TUG cog (*p* = 0.04), uncorrected for multiple comparisons. A 1% increase in caudate nucleus volume is estimated to decrease the TUG cog scores by 0.45 units and the 95% adjusted confidence interval for this change is (−1.09, 0.18). Gray matter volume in cerebellum was significantly associated with TUG alone (Table 2, *p* = 0), with higher gray matter volume associated with worse performance on the TUG. A 1% increase in cerebellum volume is estimated to increase the TUG alone scores by 0.26 and the 95% adjusted confidence interval for this change is (0.02, 0.50).

**Figure 2:**
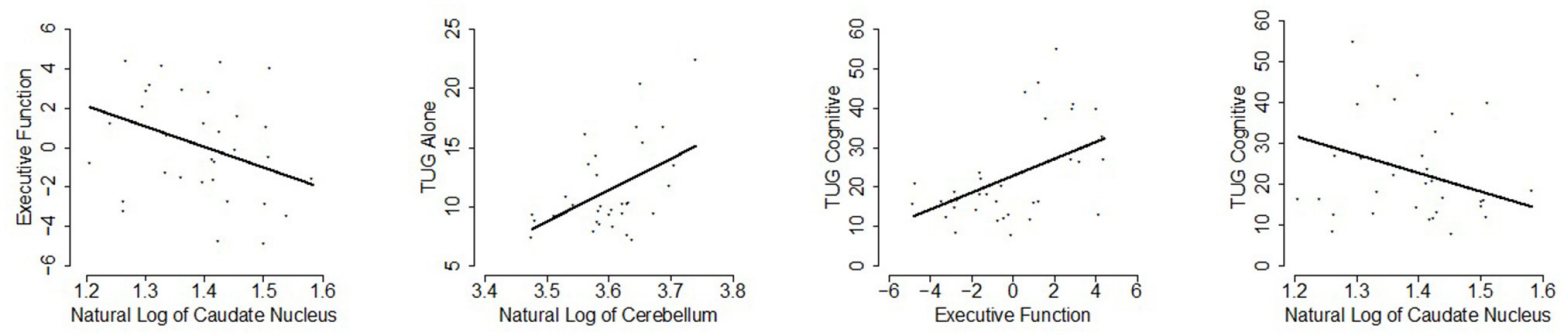
**Scatterplots of statistically significant relationships (***p*** ≤ 0.05; uncorrected for multiple testing) are shown with the linear regression line of the predictor vs. response at mean age overlaid**.

Additionally, an association emerged for a relationship between gray matter volume in the caudate and the executive function, such that higher caudate volume was correlated with stronger executive function (*p* = 0.05), uncorrected for multiple comparisons. A 1% increase in caudate nucleus volume is estimated to decrease the executive function composite score by 0.10 and the 95% adjusted confidence interval for this change is (−0.25, 0.04). These results are presented in Table 2 and significant relationships are displayed in scatterplots in Figure 2.

## Discussion

In this investigation of mobility and brain abnormalities in older adults with cognitive impairment, we found relationships between brain volume and mobility limitations. Specifically, higher caudate nucleus volume was associated with faster TUG cog speed. In addition, larger gray matter volumes in cerebellum were associated with poorer performance on the TUG alone. By contrast, we found little evidence of association between white matter hyperintensities and mobility performance. Taken together, our data provide evidence that gray matter abnormalities in subcortical brain regions, rather than WMH burden or cortical gray matter volume reduction, are associated with mobility performance in the context of cognitive impairment, i.e., for MCI and AD patient populations.

Our main hypothesis of associations between brain volume and mobility performance in cognitively impaired older adults was supported. Most notably, larger gray matter volume in the caudate nucleus was associated with faster speed on a functional mobility task. The basal ganglia's contribution to movement is well-established in cognitively healthy individuals and in populations with targeted basal ganglia lesions. Our findings suggest that in cognitively impaired individuals, smaller gray matter volume in basal ganglia may be associated with mobility limitations. In the present study, this relationship was evident across a range of cognitive decline, from MCI to dementia.

Additionally, an association emerged suggesting a positive relationship between caudate volume and executive function performance. The caudate nucleus' role in executive functioning is well-documented by others (Alexander et al., 1986; Brown et al., 1997; Packard and Knowlton, 2002; Elliott, 2003), with deficits in attention, working memory, inhibition, and initiation described in conjunction with smaller caudate volume and reduced connectivity. Functional imaging data has largely corroborated the model of functional cortico-striatal connectivity (e.g., Postuma and Dagher, 2006). Caudate and frontal cortices are thought to work in concert to support the expression of executive function (Bonelli and Cummings, 2007). Given this association, it is possible that among individuals with MCI and Alzheimer's disease, reduced striatal volume may impact mobility as a result of declines in cognition. Alternatively, reductions in speed of movement may contribute to impaired executive function.

Our finding of an inverse association between cerebellum volume and mobility performance is surprising. Evidence from cognitively healthy individuals suggests links between decreased cerebellar volumes and slow gait, and data from cognitively impaired individuals demonstrate associations between diminished cerebellar gray matter and gait dysfunction (Olazarán et al., 2013; Colloby et al., 2014). By contrast, one study found increases in cerebellar volume associated with positive *APOEe*4 status (Alexander et al., 2012), suggesting a similar relationship between disease risk and cerebellar volume as demonstrated in our analysis. To our knowledge, our findings are the first to document a positive association between increased cerebellar volume and slower mobility. These findings are preliminary and require further validation.

In contrast to associations between gray matter volume and mobility, our hypothesis that total white matter lesion volume would be associated with mobility performance was not supported. As a result, it was not possible to evaluate whether cognition would mediate such a relationship. Similarly, results have been mixed in the falls literature regarding associations between white matter and falls. While some researchers have found that white matter hyperintensities were correlated with greater falls risk (Blahak et al., 2009; Zheng et al., 2011), others have found no association (Baloh et al., 2003). However, as noted below, it is possible that due to specific inclusion criteria, our sample may have displayed a restricted range of white matter lesion burden, thereby constraining observation of significant possible relationships between these variables. Additionally, the lack of association between white matter lesions and mobility in our sample may have resulted from low sample size. Further research is needed to explore the relationships between white matter, and mobility.

Finally, consistent with evidence from our group and others (Doi et al., 2014; Fischer et al., 2014; Montero-Odasso et al., 2014), performance on executive function tasks was associated with time on the TUG cog. This was expected, given the contribution of working memory, divided attention and processing speed to performance on the dual-task TUG. However, without stronger evidence of a relationship between cortical brain volume and cognition, we can only speculate on the role cognitive function may play in mediating mobility limitations associated with structural brain abnormalities. These relationships may have been obscured by the presumed global deterioration in our cognitively impaired sample.

## Strengths/limitations

Strengths of the study include its comprehensiveness. The evaluation of WMH, regional gray matter volumes, executive function, and mobility were examined concomitantly. Moreover, the cognitive status of participants was well-characterized as either MCI or AD by a multi-disciplinary team. The examination of relationships between mobility and brain structures within cognitively impaired populations is critical to understanding mechanisms contributing to mobility limitations; it cannot be assumed that the relationships occurring in cognitively healthy individuals will be unchanged among those with cognitive impairment. For example, the relationship between gray and white matter volumes might be altered within the context of Alzheimer's disease pathology. These relationships require ongoing study among individuals with MCI and AD.

Limitations include the small and selected sample size. Further, as we excluded individuals with significant movement problems, we may have selected a sub-set of fallers with lower WMH volume. Thus, our study may have been under-powered or too restricted in range to accurately detect relationships between WMH and mobility and falls. Because we intended the project to be exploratory, we present analyses with and without correction for multiple comparisons. This sample was recruited from the Alzheimer's Disease Research Center, raising the possibility of selection bias. Individuals from this sample may be different from the underlying population base, and replication of these results will be important (Kukull and Ganguli, 2012). Additional variables of interest related to physical function in aging (such as sleep, medications, and cardiovascular health) may also play a role in mobility and falls in MCI and AD. Finally, the study design was cross sectional; future studies will benefit from utilizing a prospective design to record falls.

## Conclusions

To conclude, among cognitively impaired patients, gray matter volumes in caudate nucleus and cerebellum were implicated in mobility performance. By contrast, the role of WMH burden in mobility was negligible. These findings suggest that subcortical gray matter structures may be particularly important for mobility among individuals whose cognition has begun to decline, and highlight the role of subcortical regions in the inter-related systems of brain, cognition, and mobility.

## Ethics statement

The study was approved by the University of Wisconsin-Madison IRB, and was conducted in compliance with guidelines on human experimentation.

## Author contributions

BF contributed to data analysis, results interpretation, drafting, and revising paper; RB contributed to data and statistical analyses, interpretation, drafting, and revising paper; BB contributed to data acquisition and analysis, interpretation, and revising paper; AB contributed to imaging analysis and interpretation, data analysis, revising paper; ML and SH contributed to imaging analysis and interpretation, revising paper; RC contributed to statistical analysis, interpretation, and revising paper; JM contributed to data analysis and interpretation, revising paper; CG contributed to research concept, research administration, data analysis, interpretation, drafting, and revising paper. Research concept: CG; Research administration: CG; Interpretation of Results: BF, CG, JM, BB, RB, AB, RC; Statistical analyses: RB, RC; Imaging analyses: BB, AB, SH, ML; Drafting/Revising Paper: BF, RB, JM, CG, SH, ML.

### Conflict of interest statement

The authors declare that the research was conducted in the absence of any commercial or financial relationships that could be construed as a potential conflict of interest.
