# Supplementary material for: An Examination of Brain Abnormalities and Mobility in Individuals with Mild Cognitive Impairment and Alzheimer's Disease
Source: Front Aging Neurosci. 2017 Apr 5;9:86. doi: 10.3389/fnagi.2017.00086 (PMC5380746; doi:10.3389/fnagi.2017.00086)
Supplement: Supplementary file 1 [file Table1.DOCX]

**SUPPLEMENTAL MATERIAL**

TIMED UP AND GO (TUG)

1. Begin the test with the subject sitting correctly (hips all of the way to the back of the seat) in a chair with arm rests. The chair should be stable and positioned such that it will not move when the subject moves from sit to stand. The subject is allowed to use the arm rests during the sit – stand and stand – sit movements.
2. Place a piece of tape or other marker on the floor 3 meters away from the chair so that it is easily seen by the subject.
3. Instructions: “On the word GO you will stand up, walk to the line on the floor, turn around and walk back to the chair and sit down. Walk at your regular pace.
4. Start timing on the word “GO” and stop timing when the subject is seated again correctly in the chair with their back resting on the back of the chair.

TIMED UP AND GO with COGNITIVE TASK (TUGCOG)

1. The subject completes the TUG while reciting the days of the week in backwards order.

DYNAMIC GAIT INDEX (DGI)

**1. Gait level surface _____**

Instructions: Walk at your normal speed from here to the next mark (20')

Grading: Mark the lowest category that applies.

(3) Normal: Walks 20', no assistive devices, good sped, no evidence for imbalance, normal gait pattern

(2) Mild Impairment: Walks 20', uses assistive devices, slower speed, mild gait deviations.

(1) Moderate Impairment: Walks 20', slow speed, abnormal gait pattern, evidence for imbalance.

(0) Severe Impairment: Cannot walk 20' without assistance, severe gait deviations or imbalance.

**2. Change in gait speed _____**

Instructions: Begin walking at your normal pace (for 5'), when I tell you “go,” walk as fast as you can (for 5'). When I tell you “slow,” walk as slowly as you can (for 5').

Grading: Mark the lowest category that applies.

(3) Normal: Able to smoothly change walking speed without loss of balance or gait deviation. Shows a significant difference in walking speeds between normal, fast and slow speeds.

(2) Mild Impairment: Is able to change speed but demonstrates mild gait deviations, or not gait deviations but unable to achieve a significant change in velocity, or uses an assistive device.

(1) Moderate Impairment: Makes only minor adjustments to walking speed, or accomplishes a change in speed with significant gait deviations, or

speed but has significant gait deviations, or changes speed but loses balance but is able to recover and continue walking.

(0) Severe Impairment: Cannot change speeds, or loses balance and has to reach for wall or be caught.

**3. Gait with horizontal head turns _____**

Instructions: Begin walking at your normal pace. When I tell you to “look right,” keep walking straight, but turn your head to the right. Keep looking to the right until I tell you, “look left,” then keep walking straight and turn your head to the left. Keep your head to the left until I tell you “look straight,“ then keep walking straight, but return your head to the center.

Grading: Mark the lowest category that applies.

(3) Normal: Performs head turns smoothly with no change in gait.

(2) Mild Impairment: Performs head turns smoothly with slight change in gait velocity, i.e., minor disruption to smooth gait path or uses walking aid.

(1) Moderate Impairment: Performs head turns with moderate change in gait velocity, slows down, staggers but recovers, can continue to walk.

(0) Severe Impairment: Performs task with severe disruption of gait, i.e., staggers outside 15” path, loses balance, stops, reaches for wall.

**4. Gait with vertical head turns _____**

Instructions: Begin walking at your normal pace. When I tell you to “look up,” keep walking straight, but tip your head up. Keep looking up until I tell you, “look down,” then keep walking straight and tip your head down. Keep your head down until I tell you “look straight,“ then keep walking straight, but return your head to the center.

Grading: Mark the lowest category that applies.

(3) Normal: Performs head turns smoothly with no change in gait.

(2) Mild Impairment: Performs head turns smoothly with slight change in gait velocity, i.e., minor disruption to smooth gait path or uses walking aid.

1) Moderate Impairment: Performs head turns with moderate change in gait velocity, slows down, staggers but recovers, can continue to walk.

(0) Severe Impairment: Performs task with severe disruption of gait, i.e., staggers outside 15” path, loses balance, stops, reaches for wall.

**5. Gait and pivot turn _____**

Instructions: Begin walking at your normal pace. When I tell you, “turn and stop,” turn as quickly as you can to face the opposite direction and stop.

Grading: Mark the lowest category that applies.

(3) Normal: Pivot turns safely within 3 seconds and stops quickly with no loss of balance.

(2) Mild Impairment: Pivot turns safely in > 3 seconds and stops with no loss of balance.

(1) Moderate Impairment: Turns slowly, requires verbal cueing, requires several small steps to catch balance following turn and stop.

(0) Severe Impairment: Cannot turn safely, requires assistance to turn and stop.

**6. Step over obstacle ____**

Instructions: Begin walking at your normal speed. When you come to the shoebox, step over it, not around it, and keep walking.

Grading: Mark the lowest category that applies.

(3) Normal: Is able to step over the box without changing gait speed, no evidence of imbalance.

(2) Mild Impairment: Is able to step over box, but must slow down and adjust steps to clear box safely.

(1) Moderate Impairment: Is able to step over box but must stop, then step over. May require verbal cueing.

(0) Severe Impairment: Cannot perform without assistance.

**7. Step around obstacles _____**

Instructions: Begin walking at normal speed. When you come to the first cone (about 6' away), walk around the right side of it. When you come to the second cone (6' past first cone), walk around it to the left.

Grading: Mark the lowest category that applies.

(3) Normal: Is able to walk around cones safely without changing gait speed; no evidence of imbalance.

(2) Mild Impairment: Is able to step around both cones, but must slow down and adjust steps to clear cones.

(1) Moderate Impairment: Is able to clear cones but must significantly slow, speed to accomplish task, or requires verbal cueing.

(0) Severe Impairment: Unable to clear cones, walks into one or both cones, or requires physical assistance.

**8. Steps _____**

Instructions: Walk up these stairs as you would at home, i.e., using the railing if necessary. At the top, turn around and walk down.

Grading: Mark the lowest category that applies.

(3) Normal: Alternating feet, no rail.

(2) Mild Impairment: Alternating feet, must use rail.

(1) Moderate Impairment: Two feet to a stair, must use rail.

(0) Severe Impairment: Cannot do safely.

**TOTAL SCORE: ___ / 24**

|  | Total  Mean (SD) | MCI  Mean (SD) | AD  Mean (SD) |
| --- | --- | --- | --- |
| **N** | 34 | 17 | 17 |
| HVLT-R Imm. Rec. | 15.2 (5.8) | 17.4 (5.7) | 13.1 (5.2) |
| HVLT-R Del. Rec. | 2.2 (2.8) | 3.8 (2.9) | 0.6 (1.7) |
| HVLT-R Recog. Cor. | 8.7 (3.0) | 9.9 (1.7) | 7.5 (3.5) |
| HVLT-R Recog. Disc. | 3.4 (2.6) | 2.9 (1.8) | 3.9 (3.2) |
| BVMT-R Imm. Rec. | 8.0 (5.4) | 10.8 (5.5) | 5.2 (3.6) |
| BMVT -R Del. Rec. | 2.6 (2.7) | 4.2 (2.8) | 1.1 (1.3) |
| BMVT-R Recog. Cor. | 4.8 (1.2) | 5.1 (0.9) | 4.5 (1.4) |
| BMVT-R Recog. Disc. | 1.3 (1.9) | 0.7 (1.1) | 1.9 (2.2) |
| HVLT-R = Hopkins Verbal Learning Test-Revised; BVMT-R = Brief Visual Memory Test-  Revised; Imm. Rec. = Immediate Recall; Del. Rec = Delayed Recall; Recog. Cor. = Recognition Correct; Recog. Disc. = Recognition Discrimination  Supplementary Table 2  Participant Characteristics   \|  \| Total  Mean (SD) \| MCI  Mean (SD) \| AD  Mean (SD) \| \| --- \| --- \| --- \| --- \| \| N \| 34 \| 17 \| 17 \| \| Progressive Romberg total \| 2.2 (0.8) \| 2.2 (0.9) \| 2.1 (0.8) \| \| Injuries from a fall (N, %) \| 5, 16.7% \| 2, 13.3% \| 3, 20% \| \| Visual Equivalent \| 20/28 (/10.6) \| 20/26 (/11.2) \| 20/29 (/10.1) \| \| Total Cholesterol \| 191.5 (36.7) \| 194.9 (41.3) \| 187.7 (31.7) \| \| Smoker (N, %) \| 2, 5.9% \| 1, 5.9% \| 1, 5.9% \| \| Blood pressure medication (N, %) \| 20, 62.5% \| 8, 53.3% \| 12, 70.6% \| \| Diabetes (N, %) \| 3, 8.8% \| 3, 17.6% \| 0, 0.0% \| \| Depression (N, %) \| 11, 37.9% \| 6, 42.9% \| 5, 33.3% \| | | | |

Supplementary Table 1

**Memory Scores**
